# A Room Temperature Nitric Oxide Gas Sensor Based on a Copper-Ion-Doped Polyaniline/Tungsten Oxide Nanocomposite

**DOI:** 10.3390/s150407084

**Published:** 2015-03-24

**Authors:** Shih-Han Wang, Chi-Yen Shen, Jian-Ming Su, Shiang-Wen Chang

**Affiliations:** 1 Department of Chemical Engineering, I-Shou University, Kaohsiung 84001, Taiwan; E-Mails: shwang@isu.edu.tw (S.-H.W.); y10171019@yahoo.com.tw (S.-W.C.); 2 Department of Electrical Engineering, I-Shou University, Kaohsiung 84001, Taiwan; E-Mail: z560327@yahoo.com.tw

**Keywords:** copper-ion-doped polyaniline/tungsten oxide nanocomposite, nitric oxide (NO), gas sensor, Rayleigh surface acoustic wave (SAW)

## Abstract

The parts-per-billion-level nitric oxide (NO) gas sensing capability of a copper-ion-doped polyaniline/tungsten oxide nanocomposite (Cu^2+^/PANI/WO_3_) film coated on a Rayleigh surface acoustic wave device was investigated. The sensor developed in this study was sensitive to NO gas at room temperature in dry nitrogen. The surface morphology, dopant distribution, and electric properties were characterized using scanning electron microscopy, energy-dispersive X-ray spectroscopy mapping, and Hall effect measurements, respectively. The Cu^2+^/PANI/WO_3_ film exhibited high NO gas sensitivity and selectivity as well as long-term stability. At 1 ppb of NO, a signal with a frequency shift of 4.3 ppm and a signal-to-noise ratio of 17 was observed. The sensor exhibited distinct selectivity toward NO gas with no substantial response to O_2_, NH_3_ and CO_2_ gases.

## 1. Introduction

The demand for highly sensitive sensors featuring selectivity toward specific gases in various applications, such as industrial process control, environment protection, food safety, healthcare, and security, has increased. Several sensing technologies, including optical, electronic, and electrochemical methods, have been developed for use in various applications [1,2,3]. Nitric oxide (NO) emissions are mainly generated through the combustion of fossil fuels, and combustion that occurs in power plants and vehicle engines. NO sensors are required to differentiate NO from other combustion gases in aftertreatment systems of the waste [4]. In environmental monitoring, NO sensors operate at sub-parts-per-million levels.

NO sensors are also required for diagnosing respiratory diseases because the NO concentration in exhaled breath can indicate inflammation of the airways [5]. Studies have reported that an increased NO concentration in exhaled air is a key symptom of asthma [6,7,8]. Regarding breath analysis, sensors must be able to detect NO concentrations in the parts-per-billion range. The most common device applied in breath monitoring is the bulky chemiluminescence analyzer [9]. A sensor array based on a *yttria-stabilized zirconia* (YSZ) electrolyte and containing a WO_3_ sensing electrode and Pt-zeolite/Pt as the reference electrode designed for parts-per-billion-level NO detection was developed [10] and tested in a temperature range of 400–600 ^o^C. An NO-selective sensor based on mixtures of WO_3_ and Cr_2_O_3_ was designed for operation at a low parts-per-billion level and was operated at 300 °C [11].

As mentioned previously, metal oxides are effective materials applied in parts-per-billion-level NO detection. Among these materials, WO_3_, ZnO, and SnO_2_ are often applied in NO_x_ sensing [2,12,13,14]. However, such sensors must be operated at a high temperature, typically above 200 °C. In addition, metal-oxide-based gas sensors exhibit several disadvantages including poor selectivity, response, and recovery times in the range of minutes or even hours as well as high power consumption. In a previous study, we combined a conducting polymer, polyaniline (PANI), and a metal oxide, PANI/WO_3_ [15], to improve the sensitivity of detection at room temperature and retain the advantages of the constituent parts, which feature high surface functions to chemisorption for gas detection. Nevertheless, the detection limit for the NO gas was 23 ppb, which is insufficient for certain biomedical applications. To increase the sensitivity and lower the detection limit, selective reductive catalysts were introduced. Numerous transition metals, such as Fe, V, Cr, and Cu, exhibit low-temperature catalytic activity [16], and copper is among the most typical catalysts applied in selective catalytic reduction reactions.

This study focused on Cu^2+^/PANI/WO_3_-nanocomposite-coated surface acoustic wave (SAW) sensors featuring sensitivity and selectivity toward NO in the parts-per-billion range at room temperature in dry nitrogen. The advantages of SAW devices, such as a small size, fabrication reproducibility, and fast output, make them suitable for application in gas detection [17,18,19]. SAW sensors can respond to targets that contribute to perturbation in SAW wave propagation. Mass loading, the electric effect, and the acoustoelectric effect perturb SAW propagation while the SAW sensor detects the target gas. The change in SAW propagation can be evaluated by measuring the frequency shift and resistance change of the SAWs. Therefore, this study investigated the detection properties of a sensor by analyzing SAW responses.

## 2. Experimental Section

### 2.1. Materials and Reagents

An aniline monomer was purchased from ACROS (Bergen County, NJ, USA) and was distilled prior to use. Tungsten hexachloride (WCl_6_, Aldrich, St Paul, MN, USA), ammonium persulphate ((NH_4_)_2_S_2_O_8_, Showa, Tokyo, Japan), hydrogen chloride (HCl, Union Chemical Works Ltd., Hsinchu, Taiwan), isopropanol (TEDIA, Fairfield, OH, USA), copper sulfate (CuSO_4_, Aldrich), and ammonium hydroxide (NH_4_OH, TEDIA, Fairfield, OH, USA) were used without treatment. All chemicals used were of analytical reagent grade. 

### 2.2. Preparation of Cu^2+^/PANI/WO_3_ Films

The procedure used to produce the tungsten oxide and tin oxide/PANI solutions was similar to that employed in our previous studies [20,21,22]. Tungsten oxide gel was first synthesized and then mixed with an aniline precursor. Subsequently, tungsten (VI) hexachloride (3 g) was mixed with isopropanol (50 mL) in an ice bath. Hydrolysis was achieved by adding a 0.5 M NH_4_OH aqueous solution (5 mL) at room temperature. The chloride ion in the solution was removed using deionized water. The precipitate was peptized by slowly applying NH_4_OH and refluxed for 3 days until it become gel. Distilled aniline (0.1 mol) was dissolved in 1 M HCl and mixed with a tungsten oxide gel through vigorous stirring. An ammonium persulphate solution (0.1 M) was added dropwise into the thoroughly mixed solution and polymerization proceeded at an ambient temperature for 20 h. Subsequently, the obtained PANI/WO_3_ precipitate was rinsed using deionized water and then washed with a 1 M HCl solution. The obtained solution was diluted using i-propanol to facilitate thin film deposition.

The obtained PANI/WO_3_ solution was mixed with a CuSO_4_ aqueous solution to form a 0.05 wt % copper-ion-doped (Cu^2+^) PANI/WO_3_ film. The surface morphology and distribution of the elements were characterized using a field-emission scanning electron microscope (FE-SEM, Hitachi 4700, Toronto, Canada) and energy-dispersive X-ray spectroscopy (EDS) mapping (Horiba, Kyoto, Japan). The electric properties of the sensing film were evaluated using a Hall effect measurement system (HMS-3000, Ecopia, Chandler Heights, AZ, USA).

### 2.3. Fabrication of Surface Acoustic Wave Sensors

The SAW sensor was designed as a two-port resonator and was fabricated on a ST-cut quartz substrate. ST-cut quartz exhibits high temperature stability at room temperature [23,24,25]. Aluminum (Al) interdigital transducers (IDTs) were produced by conducting radio-frequency sputtering by using the lift-off method and exhibited a thickness of 3200 Å. The IDTs exhibited a pair number of 104, a period *p* of 32 μm, and an aperture of 960 μm. Each reflector in the device had 150 Al strip gratings; the metallization ratio was 0.5, and the length of the cavity was 605 μm. The oscillation frequency was measured by the spectrum analyzer (4395A, Agilent, Santa Clara, CA, USA) and was stable at 98.47 MHz after SAW resonator was connected with the oscillator [20]. The Cu^2+^/PANI/WO_3_ sensitive layer was spin coated on the space between the input and output IDTs and covered an area of 1.5 × 0.5 mm^2^. The thickness of the sensitive layer was approximately 3000 Å measured by ellipsometry.

### 2.4. Surface Acoustic Wave Sensor Measurement

**Figure 1:**
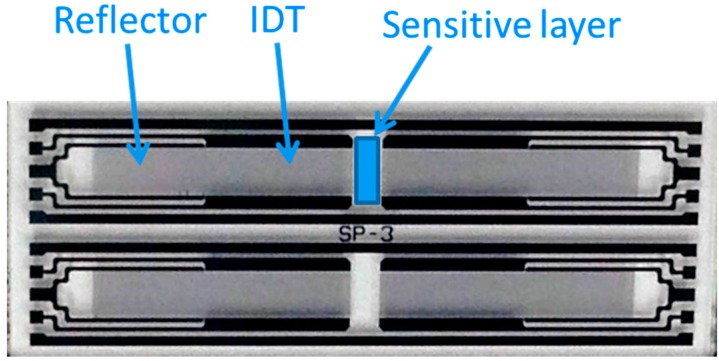
Photograph of a dual-device configuration.

**Figure 2:**
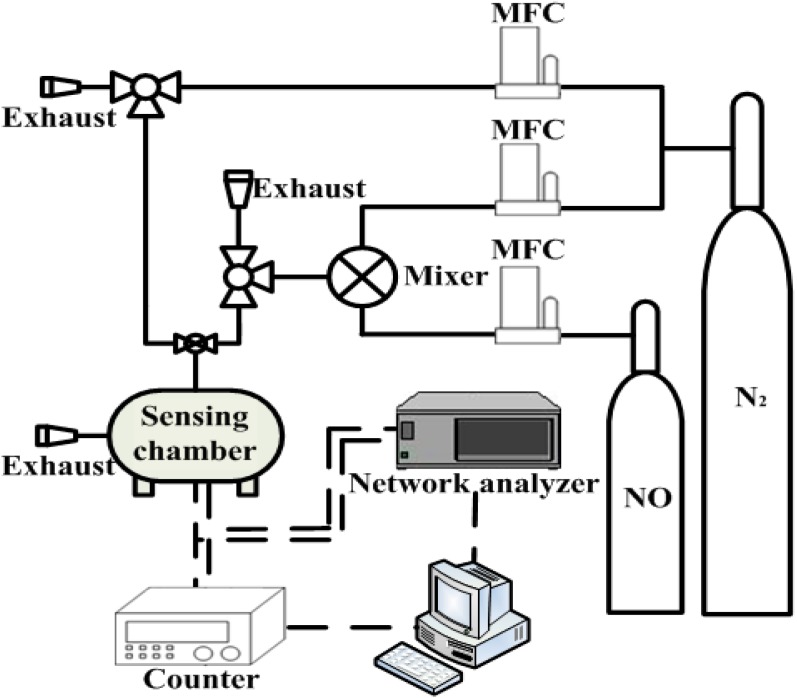
Experimental setup for measuring NO gas.

This study made use of a dual-device configuration shown in Figure 1 to reduce interference from the environment. A coated SAW resonator was used as a sensor, and a non-coated resonator was used as a reference. The dimensions of each device were 15.4 × 5.8 × 0.5 mm^3^. Figure 2 shows the setup applied in NO gas measurement experiments. High-purity nitrogen gas and certified 116 ppb and 300 ppb NO gas (Jing-De Gas Co., Kaohsiung, Taiwan) were mixed in various ratios by using a commercial gas mixer (Jing-De Gas Co.). Various concentrations of NO flowed from the gas mixer. The outflow, which was adjusted using mass flow controllers (Sierra, Kyoto, Japan), exhibited a constant flow rate of 110 mL/min. The NO breath sensor (FENO, Bedfont, UK) is an independent analytical instrument to analyze NO gas concentration generated by the gas mixer. The dual-device sensing system was placed in a temperature-stabilized, sealed 5-cm^3^ test chamber. A temperature controller maintained a constant temperature of 23 °C. The performance of the fabricated SAW sensor was measured at various concentrations of NO gas in the closed test chamber.

Two electronic configurations [26] for characterizing the SAW sensor were used in this study. One is the feedback-loop oscillator design [27] which was applied to design the SAW resonator stabilized oscillators to provide a single-frequency signal, measured by a frequency counter (53132A, Agilent, Santa Clara, CA, USA). The other one is a network analyzer (E5061B, Agilent), with software (ATeam Scientific Ltd., Hsinchu, Taiwan), connected to the SAW resonator to obtain the impedance characteristics of the sensor. A frequency counter and a network analyzer were connected to a computer system by using a GPIB interface board and used to record the responses of the sensor. The impedance measurement was carried out at the resonant frequency in this study. Noise measurement was conducted using data collected for 5 min at 20 points per minute, and noise was employed as the standard deviation of the residuals of the linear least squares fit through the data.

## 3. Results and Discussion

**Figure 3:**
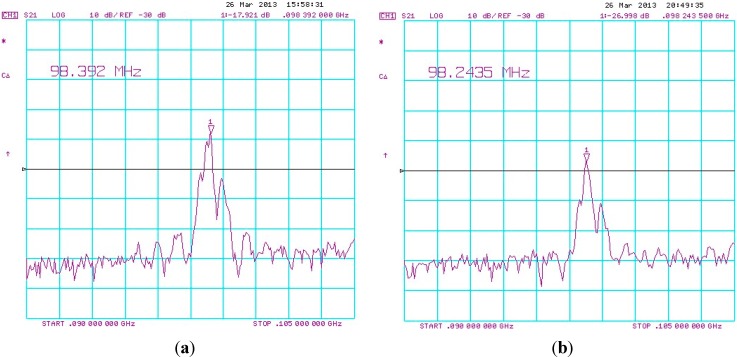
Example of frequency responses of (**a**) a raw SAW resonator and (**b**) after Cu^2+^/PANI/WO_3_ layer coating.

The operating frequency of the SAW resonator was measured before and after the Cu^2+^/PANI/WO_3_ sensitive layer was deposited and the frequencies were 98.392 MHz and 98.243 MHz, respectively, as shown in Figure 3. This negative frequency shift was accompanied by an increase in attenuation caused by mass loading on the SAW device. The same coating was applied to three sensors. The difference in the frequency shift between the sensors was less than 2%, indicating that the deposition method was reproducible.

**Figure 4:**
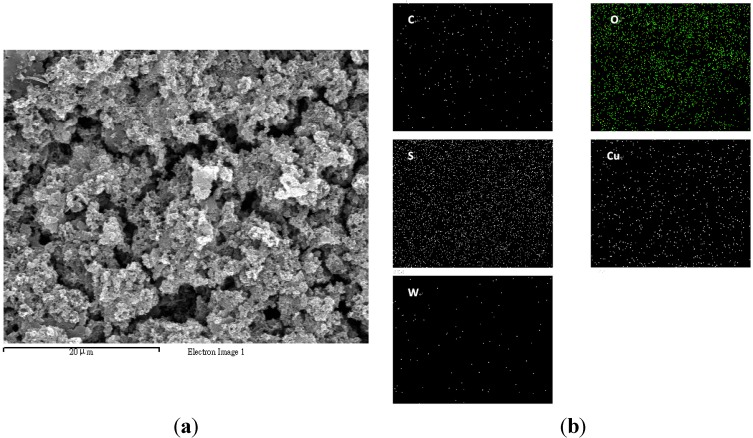
(**a**) SEM image of the as-synthesized sensitive layer and (**b**) the EDS mapping of various elements.

The surface morphology of the as-synthesized sensitive film was analyzed using a FE-SEM and the distribution of various elements was characterized using EDS mapping as shown in Figure 4a,b, respectively. Figure 4a shows that the sensitive layer exhibited a uniform and porous structure that provided a large surface area for gas sensing. In Figure 4b, the bright dots indicate the distribution of various elements. W and Cu mapping of the Cu^2+^/PANI/WO_3_ sensitive layer indicated that the tungsten oxide and copper ions were distributed in the network of PANI uniformly, constituting a porous and thin film.

**Figure 5:**
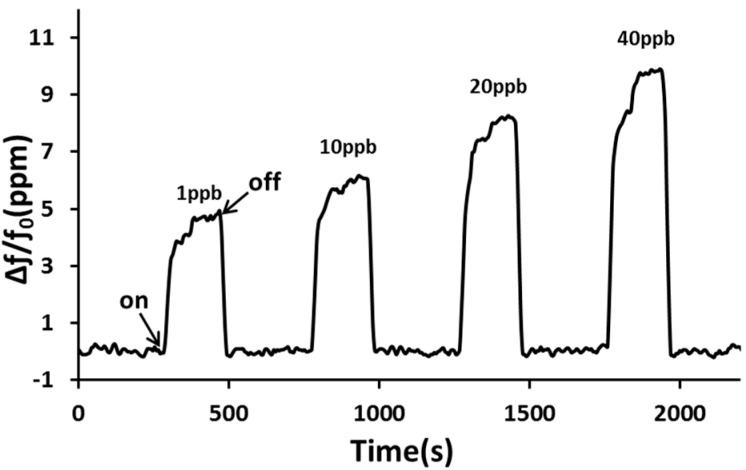
The transient response of a sensor to NO at various concentrations using dry nitrogen as carrier gas at room temperature.

The NO sensing properties in dry nitrogen were tested and the changes in frequency and resistance as a function of time were recorded when the sensor was exposed to NO gas at different concentrations. Figure 5 shows the transient response of a SAW sensor coated with a Cu^2+^/PANI/WO_3_ sensitive layer at various NO concentrations ranging from low to high. The response was calculated as Δf/f_o_ = (f – f_o_)/f_o_, where f is the resonant frequency of the sensor in the presence of NO and f_o_ is the initial resonant frequency of the sensor in dry nitrogen. Only dry nitrogen flowed through the test chamber before NO was released. After the SAW sensor coated with a Cu^2+^/PANI/WO_3_ sensitive layer was exposed to NO, it detected an increased frequency. After 180 s, NO flow was discontinued and only dry nitrogen flowed through the test chamber to enable the frequency to return to its original level. The SAW sensor coated with a Cu^2+^/PANI/WO_3_ sensitive layer exhibited a change in frequency of more than 9.6 ppm when it was exposed to NO at 40 ppb. These results indicated that an SAW sensor coated with a Cu^2+^/PANI/WO_3_ sensitive layer was able to reversibly detect parts-per-billion levels of NO at room temperature.

The frequency shift shown in Figure 5 gives a positive frequency shift during exposure to NO gas. This phenomenon can be explained by the perturbation mechanisms in Equation (1). The perturbation mechanisms of SAW propagation after absorbing gas targets can be expressed as [28,29]:

(1) $$\frac{\Delta f}{f_{o}}≅\frac{\Delta v}{v_{o}}=-c_{m}f_{o}\Delta (\frac{m}{A})+4c_{e}f_{o}\Delta (hG^{\prime })-\frac{K^{2}}{2}(\frac{1}{1+v_{o}^{2}\Delta {\left(\frac{C_{s}}{\sigma_{s}}\right)}^{2}})$$

where *c*_m_ and *c*_e_ are the coefficients of mass sensitivity and elasticity, *m*/*A* is the change in mass per unit area, *h* is thickness of the sensitive layer, G' is the shear modulus, *K*^2^ is the electromechanical coupling coefficient, σ_s_ is the sheet conductivity of the sensitive layer, C_s_ is the capacitance per unit length of the SAW substrate. The first term in Equation (1) represents the mass-loading effect, which results in a function of gas concentration. The second term is contributed from elastic properties of the sensitive layer. The third term represents the acoustoelectric effect. The frequency shift due to the change of the elastic constants of the film results in a positive change, which behaves differently when compared to the mass effect and acoustoelectric effect. Equation (1) shows a positive frequency response occurs as the elastic effect of the sensitive layer significantly increases and exceeds the change in mass and the acoustoelectric effect. All three effects were contributed to the frequency shift; however, it was not able to quantify the contribution for each in this system. Among those three effects, the elastic effect dominated the sensing response. Therefore, Figure 5 indicates that the modification of the elastic effect of the sensitive layer increased the oscillation frequency during exposure to NO gas.

**Figure 6:**
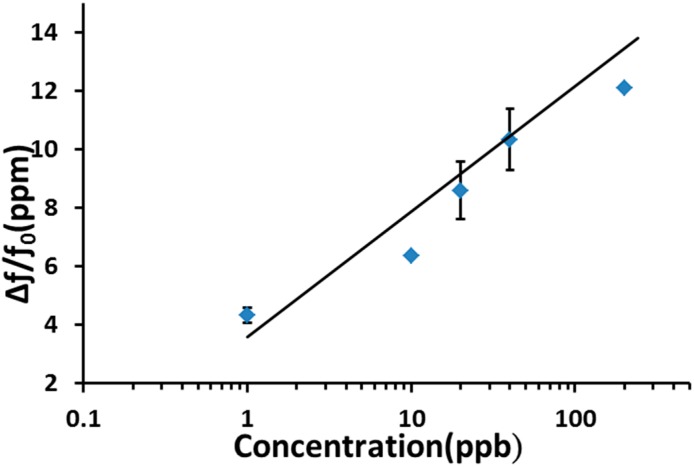
Frequency shifts of the sensor in response to various NO concentrations ranging from 1 ppb to 200 ppb using dry nitrogen as carrier gas at room temperature.

Figure 6 shows the frequency shifts of the SAW sensor coated with a Cu^2+^/PANI/WO_3_ sensitive layer at NO concentrations ranging from 1 ppb to 200 ppb in dry nitrogen. The number of adsorbed NO molecules decreased as the NO concentration decreased, and therefore, the corresponding frequency shifts decreased. At a NO concentration of 1 ppb, a signal with a frequency shift of 4.3 ppm and a signal-to-noise ratio of 17 was observed. The sensitivity of an SAW sensor is defined as the change in the frequency of the sensor in response to a change in NO concentration. The sensitivity of the sensor to the NO concentration is roughly linear in a logarithmic coordinate system.

**Figure 7:**
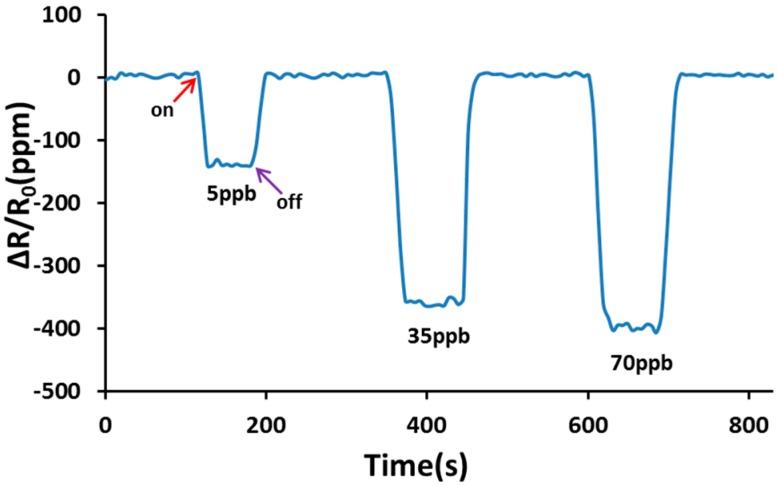
Resistance changes of the sensor at various NO concentrations using dry nitrogen as carrier gas at room temperature.

**Figure 8:**
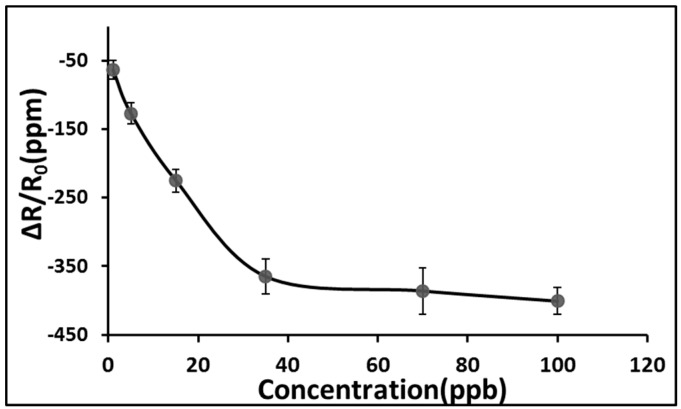
Resistance changes of the sensor at various NO concentrations using dry nitrogen as carrier gas at room temperature.

**Figure 9:**
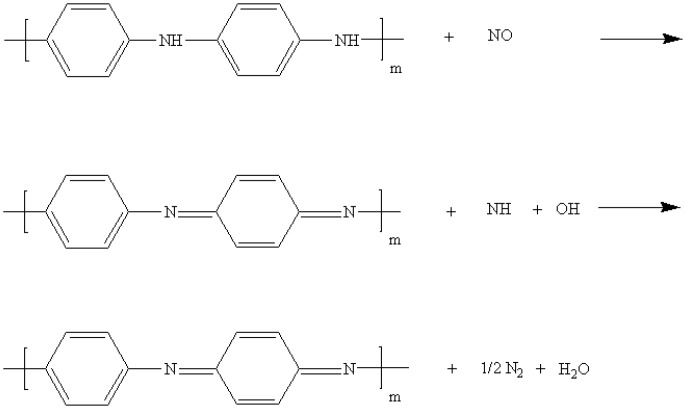
The proposed sensing mechanism.

The Cu^2+^/PANI/WO_3_ sensitive film was a p-type semiconductor measured by Hall effect measurement in this study. We also examined the NO gas sensing behavior of an SAW sensor in response to impedance changes. We observed that apparent resistance changes occurred at resonant frequency after introduction of the gas. Therefore, the impedance measurement was carried out at the resonant frequency in this study. The response is calculated as ΔR/R_o_ = (R − R_o_)/R_o_, where R is the sensor resistance in presence of NO in dry nitrogen, and R_o_ is the initial resistance of the sensor in dry nitrogen at the resonant frequency in each case. As shown in Figure 7, a decrease in resistance occurred upon exposure to NO, and the response was reversible. Figure 8 shows that the resistance changes increased according to the concentration of NO in the range of 1–100 ppb. The resistance change became gradually saturated after an NO concentration of 70 ppb. The resistance of p-type semiconductors decreases when they are exposed to oxidizing gases [30,31]. Because the Cu^2+^/PANI/WO_3_ sensitive layer was a p-type semiconductor, the resistance decreased when it was exposed to a NO atmosphere. Copper is a typical catalyst applied in selective catalytic reduction reactions, and oxygen was absented in the sensing system of this study. The reduction reaction to the NO gas occurred on the surface of the sensitive layer, and the electrons were extracted from the sensitive layer. Nitric oxide might be reduced by the sensing membrane and it might be reduced into nitrogen and water. Consequently, the resistance decreased because of the increase of the carrier concentration in the sensitive layer, as shown in Figure 7. The proposed mechanism was shown in the following Figure 9.

**Figure 10:**
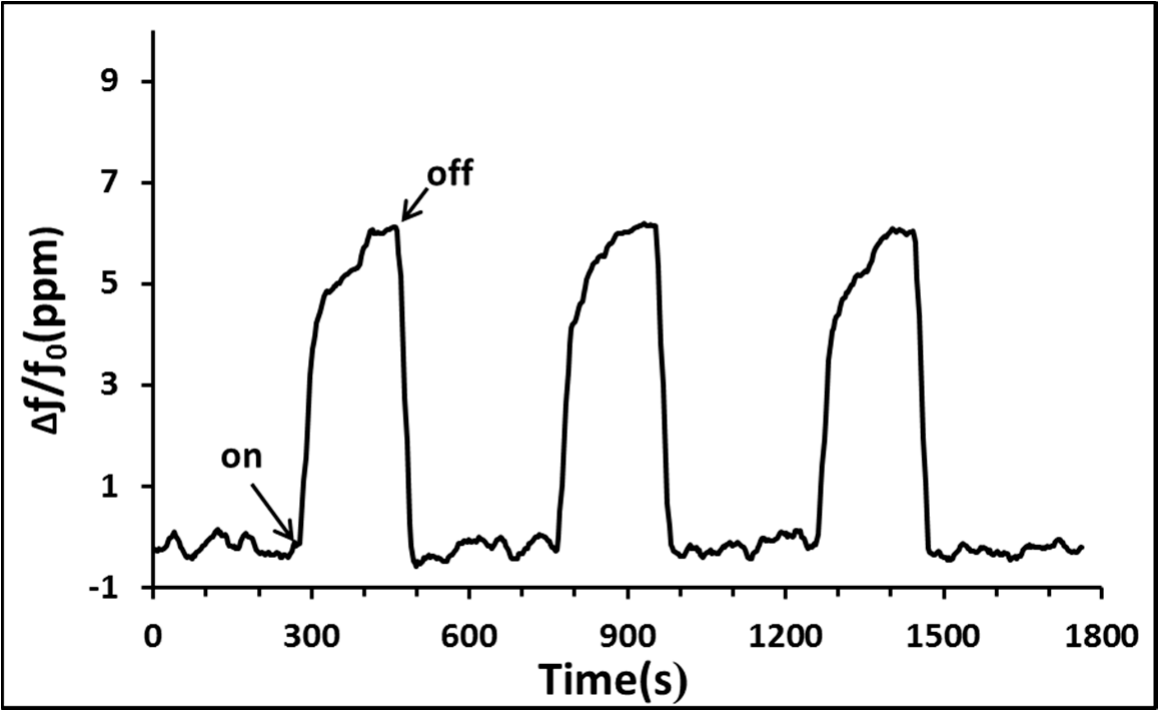
Frequency changes of the sensor at an NO concentration of 10 ppb in three gas on/off cycles using dry nitrogen as carrier gas at room temperature.

Figure 10 shows the sensor response to an NO concentration of 10 ppb in three cycles. The frequency shift was approximately 6.4 ppm in each cycle. The repeated extent of the frequency response of the SAW sensor coated with a Cu^2+^/PANI/WO_3_ sensitive layer was 92% after 27 days. 

Thus, the frequency response exhibited high repeatability and long-term stability. The response time of the sensor was calculated as the time required to reach 90% of the saturation value upon exposure to the NO gas; the recovery time was defined as the time required by the sensor to recover 90% of its saturation value after the flow of NO gas was discontinued. When the sensor was exposed to NO, an increase in frequency was observed. Figure 10 shows that the sensor responded to NO at 10 ppb, exhibiting a response time of 97 s. After NO gas flow was discontinued and the test chamber was purged with dry nitrogen, the frequency returned to its original value; the measured recovery time was 37 s.

**Figure 11:**
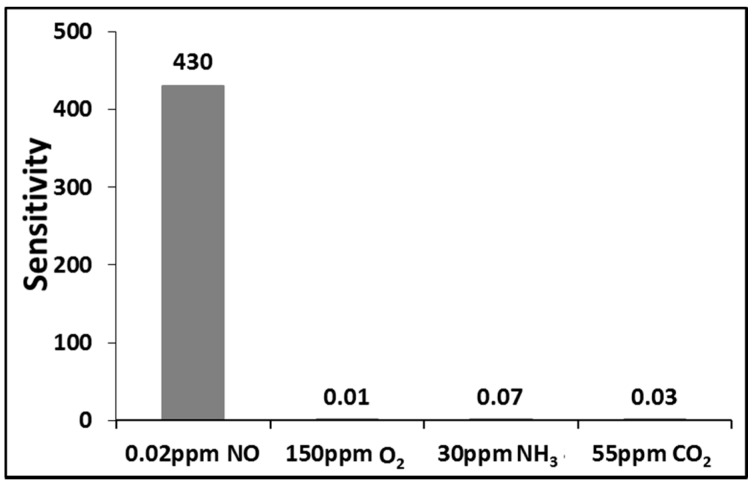
The responses of the sensor to NO gas at 20 ppb, O_2_ gas at 150 ppm, NH_3_ gas at 30 ppm, and CO_2_ gas at 55 ppm.

Figure 11 illustrates the response of the SAW sensor coated with a Cu^2+^/PANI/WO_3_ sensitive layer to NO gas at 20 ppb, O_2_ gas at 150 ppm, NH_3_ gas at 30 ppm, and CO_2_ gas at 55 ppm. The sensitivity is defined as the ratio of frequency shift to species concentration under the same gas flow rate and temperature. The gases did not affect the SAW NO sensor at the indicated concentrations. Thus, these results suggest that interference effects, such as those produced by CO_2_ and NH_3_, are negligible during the detection of NO at parts-per-billion levels in dry nitrogen.

The sensor performance has also been investigated in an atmosphere of dry air to evaluate the influence of oxygen on its response. The frequency shift to 40 ppb of NO is 9.9 ppm in dry nitrogen and 2.1 ppm in dry air, respectively. The frequency shift to 10 ppb of NO is 6.1 ppm in dry nitrogen and 0.8 ppm in dry air, respectively. It is quite clear that the sensor response is lower.

## 4. Conclusions

The results indicated that an SAW sensor coated with a Cu^2+^/PANI/WO_3_ sensitive layer exhibited high sensitivity, reversibility, repeatability, selectivity, and stability in detecting NO in dry nitrogen. At an NO concentration of 1 ppb in dry nitrogen, a signal with a frequency shift of 4.3 ppm and a signal-to-noise ratio of 17 was observed. The response and recovery times were 97 s and 37 s, respectively, at an NO concentration of 10 ppb in dry nitrogen.

Although NO easily oxidizes and becomes NO_2_ through a reaction with O_2_ in a typical environment, the NO and N_2_ gases were mainly responsible for the changes in frequency and resistance in this study because of the isolation from oxygen that the controlled chambers provided. Our preliminary findings are a promising starting point to investigate Cu^2+^/PANI/WO_3_ as sensing layer for NO gas sensing at room temperature in dry nitrogen. Further improvements of this device will be focused on optimizing the doping concentration in order to reach that the sensor will function in air and minimize the effect of environmental humidity changes on the sensor performance.
